# Re-education of the Tumor Microenvironment With Targeted Therapies and Immunotherapies

**DOI:** 10.3389/fimmu.2020.01633

**Published:** 2020-07-28

**Authors:** Shin Foong Ngiow, Arabella Young

**Affiliations:** ^1^Department of Systems Pharmacology and Translational Therapeutics, University of Pennsylvania, Philadelphia, PA, United States; ^2^Institute for Immunology, Perelman School of Medicine, University of Pennsylvania, Philadelphia, PA, United States; ^3^Department of Immunology, QIMR Berghofer Medical Research Institute, Herston, QLD, Australia; ^4^Diabetes Center, University of California, San Francisco, San Francisco, CA, United States

**Keywords:** tumor-associated myeloid cells, regulatory T cells (Tregs), natural killer T (NKT) cells, mucosal-associated invariant T (MAIT) cells, adenosine, transforming growth factor (TGF)β, prostaglandin, immune toxicity

## Abstract

The clinical success of cancer immunotherapies targeting PD-1 and CTLA-4 has ignited a substantial research effort to improve our understanding of tumor immunity. Recent studies have revealed that the immune contexture of a tumor influences therapeutic response and survival benefit for cancer patients. Identifying treatment modalities that limit immunosuppression, relieve T cell exhaustion, and potentiate effector functions in the tumor microenvironment (TME) is of much interest. In particular, combinatorial therapeutic approaches that re-educate the TME by limiting the accumulation of immunosuppressive immune cells, such as Foxp3 regulatory T cells (Tregs) and tumor-associated macrophages (TAMs), while promoting CD8^+^ and CD4^+^ effector T cell activity is critical. Here, we review key approaches to target these immunosuppressive immune cell subsets and signaling molecules and define the impact of these changes to the tumor milieu. We will highlight the preclinical and clinical evidence for their ability to improve anti-tumor immune responses as well as strategies and challenges for their implementation. Together, this review will provide understanding of therapeutic approaches to efficiently shape the TME and reinvigorate the immune response against cancer.

## Introduction

The clinical validation of key conceptual developments in the field of tumor immunology has engendered much interest in strategies to initiate immune cell function within the tumor microenvironment (TME). Central to effective anti-tumor immunity induced by cancer immunotherapies is the ability to re-educate and re-activate immune effector and cytotoxic T cells to eliminate cancer cells. As such, immunotherapies targeting T cell immune checkpoint receptors cytotoxic T-lymphocyte associated protein 4 (CTLA-4) and/or programmed death-1 (PD-1)/programmed death-ligand 1 (PD-L1) have ascended to first-line therapies for a number of solid malignancies (1). Combinatorial anti-tumor efficacy of ipilimumab (anti-CTLA-4) and nivolumab (anti-PD-1) in advanced stage melanoma and renal cell carcinoma (RCC) highlights the importance of targeting multiple immune pathways to unleash a more robust anti-tumor immune response (2, 3). In addition, FDA-approval of pembrolizumab (anti-PD-1) for the treatment of microsatellite instability-hi (MSI-h) and deficient DNA mismatch repair (dMMR) tumors, the first cancer-site agnostic treatment approval, as well as the correlation between tumor mutational burden and survival outcome sheds light on the significance of tumor genetics in initiating an immune response (4–6). Similarly, PD-L1 status has been shown to impact therapeutic outcome to PD-1/PD-L1 targeting immunotherapies (7). This highlights that a better understanding of the immune contexture and its interaction with surrounding tumor, stroma, and their derivatives (e.g., chemokines and other soluble factors) is crucial to developing novel therapeutic targets to efficiently shape and re-condition the TME, reinvigorating the immune response against cancer.

Functional anti-tumor immunity relies on both the quality (effector and cytotoxic function) and quantity (numbers and localization) of tumor-infiltrating lymphocytes (TILs) in the TME. Targeting CTLA-4 and PD-1 non-redundantly mobilizes and activates alternate T cell components, with CTLA-4 shown to inhibit priming and generation of antigen-specific T cells in the lymph nodes whereas PD-1 limits CD8^+^ T cell numbers in the tissue, for superior anti-tumor outcomes (8, 9). The concept of targeting two or more non-redundant immune regulatory pathways for enhanced anti-tumor immunity is not limited to adaptive immunity. A combination of anti-DR5, anti-CD40, and anti-CD137 agonistic antibodies aiming to induce apoptosis in tumor cells, activate antigen presenting cells (innate immunity), and co-stimulate CD8^+^ T cells (adaptive immunity), respectively, has been shown to eradicate both established transplantable and spontaneous tumors (10). Similarly, it has been shown that a combination of recombinant interleukin (IL)-2, anti-PD-1, a tumor-antigen targeting antibody, and an additional vaccine targeting three individual tumor antigens is able to eradicate a poorly immunogenic murine melanoma, via the activation of both innate and adaptive immunity (11). Here, we review key approaches to target pathways alternative to mainstream T cell checkpoint receptors to re-educate the TME and alleviate immune suppression and highlight challenges for therapy selection and implementation in the clinic.

## Improving Tumor Control With Myeloid Cells In The TME

**Figure 1:**
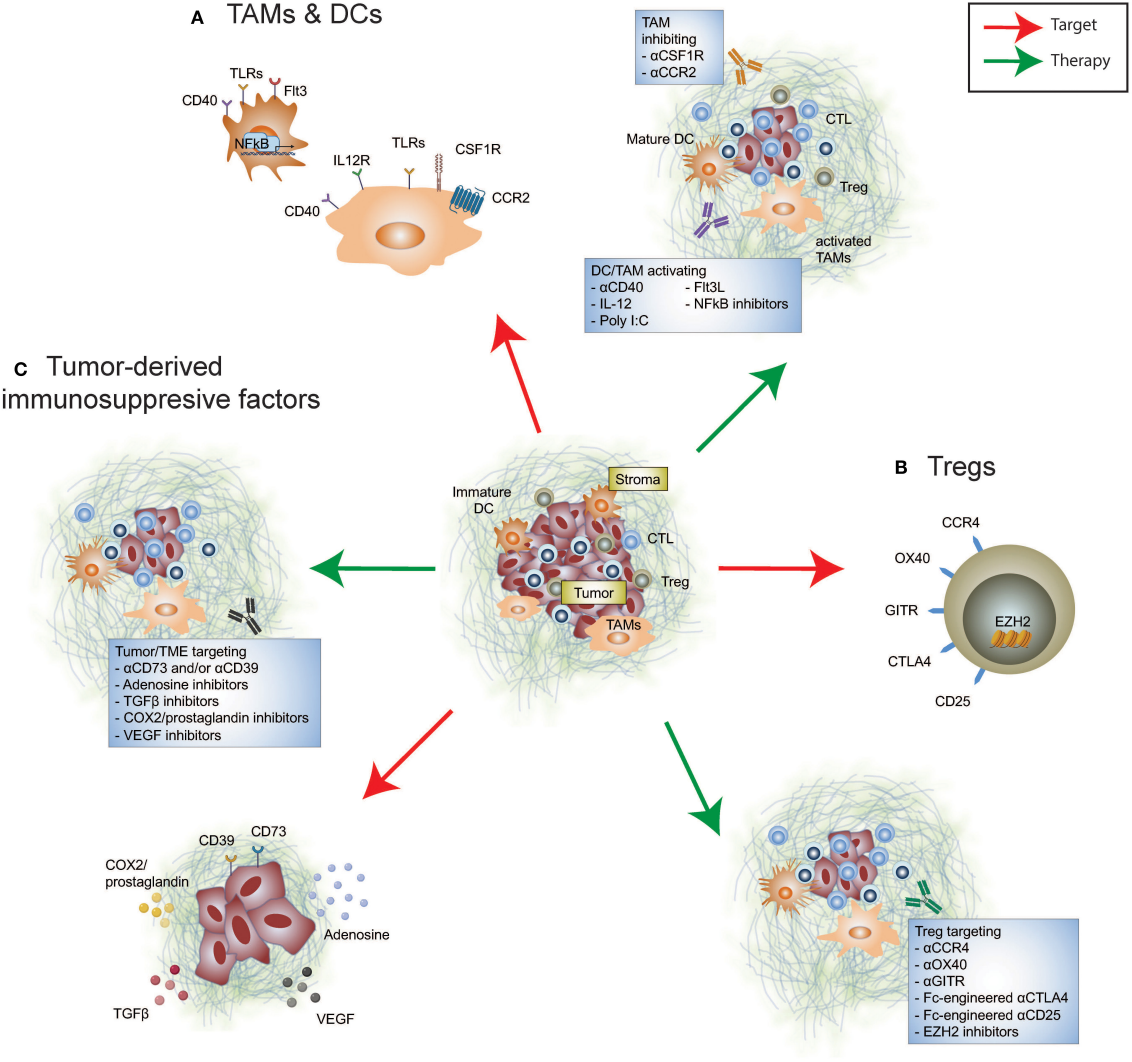
Immunosuppressive targets in the tumor microenvironment. Re-educating the tumor microenvironment to improve the response to cancer immunotherapies can be performed through targeting many cellular and immunosuppressive factors. These include **(A)** tumor-infiltrating myeloid cells such as tumor-associated macrophages and dendritic cells, **(B)** tumor-infiltrating Tregs, and **(C)** tumor-derived immunosuppressive factors. Therapeutically altering these immunomodulatory components may promote anti-tumor immunity either alone or synergize with FDA-approved immune checkpoint inhibition.

Myeloid cells predominate the TME and in many cases evolve to display an immunosuppressive phenotype and ineffective antigen presenting cells (APCs) due to the inflammatory milieu (Figure 1). Tumor-associated macrophages (TAMs) are innate immune cells of heterogeneous origins that have been shown to accumulate in the TME as tumors progress (12–14). The presence of immunosuppressive TAMs can interfere with T cell-mediated anti-tumor immune responses (15, 16). Given the absence of a universal definition for TAMs, we have listed relevant markers used in individual studies. It has been reported that an accumulation of monocyte-derived TAMs (CD11b^lo^ MHC-II^−/lo^) positively correlates with the proportion of tumor-infiltrating exhausted PD-1^+^ CD8^+^ T cells in a mouse model of mammary cancer (12), illustrating a potential mechanism by which TAMs promote tumor escape by modulating the CD8^+^ T cell response. More recently, it has been shown that TAMs (CD11b^+^ MHC-II^+^) are capable of stripping anti-PD-1 bound to PD-1^+^ T cells by binding to the antibody Fc domain, abrogating the anti-tumor activity of this immune checkpoint inhibitor (17). It remains unclear if a similar resistance mechanism also exists in the context of anti-PD-L1 therapy. However, in preclinical mouse models Fc engagement is critical for anti-PD-L1 (clone 10F.9G2) therapeutic efficacy by enabling depletion of immunosuppressive TAMs (CD11b^+^ F4/80^+^) (18). Therefore, defining the functionality of an antibodies Fc region for optimal therapeutic activity in the context of both T cells and myeloid cells is an important consideration. Additionally, modulating TAMs via targeted depletion, inhibiting active migration, and promoting activation and differentiation, as a means to re-educate the TME may increase permissiveness to immune checkpoint inhibitor therapy.

The colony stimulating factor 1 (CSF1)/CSF1 receptor (CSF1R) axis is crucial for TAM differentiation (19). Selective depletion of TAMs by targeting CSF1R using monoclonal antibodies or small molecule inhibitors has been shown to restrict CSF1R^+^ TAM accumulation in the TME, leading to reduced tumor growth in a number of mouse models (20–23). Depletion of CSF1R^+^ TAMs demonstrated efficacy in improving a wide range of existing cancer therapies, including chemotherapy, oncogene-targeted therapy, and immunotherapy (21, 23, 24). In preclinical BRAF-mutant melanoma, co-administration of PLX3397 (CSF1R inhibitor) together with PLX4720 (mutant BRAF inhibitor) effectively sensitizes a PD-1-resistant tumor model to anti-PD-1/PD-L1 therapies (23). However, CSF1R inhibition induces the expansion of polymorphonuclear myeloid-derived suppressor cells (PMN-MDSC) that may abrogate the efficacy of combination CSF1R inhibition and anti-PD-1 treatment (23, 25). Evidence that targeting CSF1R and CXCR2 signaling to inhibit TAM and PMN-MDSC expansion, respectively, alongside anti-PD-1 facilitates improved anti-tumor immune responses than either doublet combination (25). Alternatively, targeting the CCL2/CCR2 axis, a key chemokine pathway involved in macrophage migration to inflammatory sites, to limit their entry into the TME enabled numerical and functional improvement of intratumoral lymphocyte infiltrate (26–29). Wu et al. demonstrated improved survival outcomes in cutaneous T-cell lymphoma-bearing mice treated with a CCR2 inhibitor and anti-PD-1 (28). Collectively, these studies highlight that inhibition of pro-tumor TAMs in the TME reinvigorates the anti-PD-1-driven T cell response. Of note, a phase I/II clinical trial accessing the combinatorial effect of nivolumab, GVAX (a cancer vaccine expressing GM-CSF) and BMS-813160 (a CCR2/CCR5 dual antagonist) in pancreatic ductal adenocarcinoma (PDAC) is currently underway (NCT03767582).

It is worth highlighting that not all tumor-infiltrating myeloid cells promote tumor growth. The production of CXCL9 and CXCL10, predominantly by TAMs (CD11b^+^ Ly6C^int^ CD11c^+^ F4/80^+^), enhances CD8^+^ T cell infiltration and tumor control in response to combination anti-PD-1 and anti-CTLA-4 in a mouse model of mammary adenocarcinoma (30). High production of these chemokines within the TME is associated with better survival outcomes in melanoma patients receiving combination treatment (30). In light of these findings, reprogramming these innate immune cell subsets may be beneficial due to their antigen presenting properties promoting infiltration of an effective anti-tumor T cell response. In KPC (LSL-Kras^G12D/+^;LSL-Trp53^R172H/+^;Pdx-1-Cre) PDAC TAMs (CD11b^+^ Gr1^−^ F4/80^+^) enable T cell exclusion and consequently, resistance to immune checkpoint therapy is likely driven by the absence of effector T cells that can be modulated (31, 32). Independent studies of mouse pancreatic models have demonstrated the remodeling activity of the agonistic CD40 antibody by overcoming T cell exclusion in the TME, leading to improved therapeutic response to anti-CTLA-4, and/or anti-PD-1 (33–35). Using a T cell-rich but anti-PD-1 resistant mammary carcinoma model, we have recently demonstrated that IL-12 induced by an agonistic CD40 antibody could render terminally exhausted PD-1^hi^ tumor-infiltrating CD8^+^ T cells into their PD-1^int^ progenitor state (36), leading to improved anti-tumor immunity in response to anti-PD-1 following sensitization by anti-CD40 agonism.

An additional therapeutic approach to re-educate the TME and bolster the efficacy of immune checkpoint therapy is combination treatment with FMS-like tyrosine kinase 3 ligand (FLT3L) and poly I:C treatment, to expand and enhance maturation of anti-tumor CD103^+^ dendritic cells (DCs) resulting in a dramatic increase of intratumoral T cells (37). Notably, T cell-activating IL-12 producing CD103^+^ DCs diminish over time (37–41), suggesting that they may facilitate tumor control during tumor initiation. Beavis et al. also reported a role for anti-PD-1/CTLA-4 activated CD4^+^ Foxp3^−^ cells in enhancing IL-12 production by CD103^+^ DCs, which in turn promoted T cell-mediated anti-tumor immunity in mice (42). The persistence of intratumoral stimulatory DCs (CD103^+^ BDCA-3^+^) defined by gene expression profiles corelated with improved overall survival outcomes and was associated with higher TIL measurements in metastatic melanoma (43). Mediating the abundance of intratumoral stimulatory DCs was the presence of tumor-infiltrating natural killer cells and expression of FLT3L, together these components may assist in determining anti-PD-1 therapeutic response and identify therapeutic strategies to potentiate efficacy (43). More recently, a cluster of DCs named mregDCs (mature DCs enriched in immunomodulatory molecules) co-expressing immunoregulatory genes (*Cd274, Pdcd1lg2*, and *Cd200*) and maturation genes (*Cd40, Ccr7*, and *Il12b*) was found in single cell analysis of mouse and human non-small cell lung cancer (NSCLC) DC infiltrate (44). Of note, neutralizing IL-4 was shown to enhance mregDC IL-12 production, repressing lung adenocarcinoma in mice (44). With advances in high-throughput single-cell analysis to provide fine-detail of immune infiltrate in tumors, it is likely to facilitate an expansion of our repertoire of novel targets that will assist to re-educate DCs and other myeloid cells specifically in the TME.

## Re-Educating Suppressive and Unconventional T Cells In The TME

Regulatory T cells (Tregs) serve as a barrier to limit inflammation, however, their enrichment in the TME of established cancer correlates with poor prognosis and a dampened anti-tumor immune response (Figure 1). Clinical studies have resolved that a higher effector/Treg ratio is associated with favorable outcomes in multiple solid cancers (45, 46). In addition to Treg-induced suppression of effector T cells by manipulating their migration, activation, functionality and/or survival (47, 48), Tregs are able to form an immunosuppressive barrier capable of limiting the trafficking of activated antigen-specific CD8^+^ T cells into the TME (49). Importantly, Foxp3^+^ Tregs promote effector CD4^+^ and CD8^+^ TIL dysfunction, with improved cytokine-producing capacity upon Treg depletion and reinvigorated T cell responses to immune checkpoint blockade (50–52). However, systemic Treg depletion introduced transiently can still increase susceptibility of mice to autoimmunity (53), indicative that identifying an appropriate target to specifically remove intratumoral Foxp3^+^ Tregs will be advantageous for maintaining therapeutic safety.

Intratumoral Foxp3^+^ Tregs are highly suppressive, with an activated phenotype marked by the expression of several classes of immune receptors [ENTPD1 (CD39), CTLA-4, OX40, and GITR], and chemokine receptors (CCR4). Studies using preclinical mouse models showed that anti-CTLA-4 (clone 9H10, 9D9, and H11; antagonist), anti-OX40 (clone OX86; agonist) and anti-GITR (clone DTA-1; agonist) exhibited varying levels of intratumoral Treg depleting activity *in vivo* that was critical to their efficacy (54–59). Anti-CTLA-4 (clone 9D9, mouse IgG2a) and anti-OX40 were shown to specifically deplete intratumoral Tregs but not peripheral Tregs (56, 57). Tumor-infiltrating Treg depletion by anti-CTLA-4 enhanced anti-PD-1 sensitivity to the previously resistant AT3 mouse mammary carcinoma (36). In the clinic, mogamulizumab (an anti-human CCR4 antibody, engineered for ADCC activity) was developed to specifically deplete CCR4^+^ suppressive Tregs found in the TME (60), and is undergoing testing in combination with T cell checkpoint targets in Phase I/II clinical trials (NCT02301130, NCT02705105, NCT02476123, and NCT02946671). While it remains unclear whether combining anti-CCR4 and anti-PD-1 provides favorable survival benefits, an increase in the proportion of CD8^+^ T cells and a reduction in activated Foxp3^hi^ Tregs was observed in TILs from patients, along with an acceptable safety profile [(61); NCT02476123]. With advances in antibody engineering, we should expect refinement of antibodies for both existing and novel targets to modulate TME-specific Tregs to enhance anti-tumor immunity (51, 62, 63). Revisiting targeting CD25-expressing Tregs, Vargas et al. found that by altering the IgG backbone (from rat IgG1 to mouse IgG2a) greater specificity was afforded toward intratumoral Treg depleting activity by an Fc-optimized version of CD25 antibody (64). Anti-CD25-mediated intratumoral Treg depletion synergized with PD-1 blockade therapy in a number of mouse cancer models (64), highlighting the importance of remodeling the Treg dynamics within the TME to enhance checkpoint blockade therapy. Translation of this combination needs to be thoroughly examined, to limit the depletion of alternate CD25-expressing cell types including effector T cells and NK cells.

Given the critical role of Tregs in maintaining immune homeostasis, attenuating intratumoral Treg suppressive function may be a safer approach to remodel the TME while minimizing the risk of systemic autoimmunity. Studies from a series of experimental modeling showed that the disruption of Foxp3, the critical transcription factor to maintain Treg lineage, altered their suppressor function (65–67). This also resulted in the generation of pathogenic effector T cells (67, 68). However, disruption of intratumoural Treg suppressive function has been shown without the loss of its Foxp3^+^ Treg identity. Neuropilin-1 (NRP1) appears crucial to maintain intratumoural Treg stability without aberrant loss of Foxp3 identity, and anti-NRP1 displayed therapeutic efficacy in suppressing tumor growth (69). Notably, using a co-transfer model of NRP1-intact and NRP1-deficient Tregs, interferon (IFN)-γ produced by NRP1-deficient Tregs is capable of causing fragility to the suppressive capacity of NRP1-intact Tregs, resulting in improved host anti-tumor immunity (70). Similar to the role of NRP1 to maintain Treg stability, the histone H3K27 methyltransferase enhancer of zeste homolog 2 (EZH2) has been recently shown to be critical for the maintenance of activated Foxp3^+^ Tregs (71). EZH2 inhibition destabilizes Foxp3 expression and inhibits tumor growth *in vivo* (72, 73). While targeting these pathways may be able to provide an opportunity to dismantle Treg suppression within the TME, these therapies still lack specificity to this cell type. Understanding the role of these molecules in multiple cell types and disease settings is likely to dictate their applicability for utility in cancer immunity.

Besides Tregs, unconventional T cells have also received considerable interest in tumor immunology for their immunoregulatory role. In contrast to CD8^+^ and CD4^+^ T cells that interact with MHC class I and II molecules, unconventional T cells such as natural killer T (NKT) cells interact with non-classical MHC CD1d molecules (74). α-GalCer (a glycoplipid molecule derived from a marine sponge extract) is a known ligand for NKT cells, and has been widely used to experimentally modulate NKT cells (75). α-GalCer-activated NKT cells are capable of producing high levels of cytokines (including IFN-γ and IL-21), anti-tumor effector and cytotoxic molecules (perforin and granzymes), and elicit direct tumor lysing properties (76–78), which assists to alleviate immunosuppression and enhances DC maturation, leading to improved anti-tumor T cell immunity (79–81). Song et al. demonstrated that NKT cells specifically kill monocytes pulsed with neuroblastoma cell lysate and reduce tumor-infiltrating monocytes in a non-classical MHC-dependent manner (82), highlighting a role for NKT cells in shaping the immune infiltrate in the TME. Studies in mice reported superior anti-tumor activity when α-GalCer therapy to drive NKT cell activity was combined with anti-PD-1 (83, 84). However, most clinical trials assessing the anti-tumor effect of α-GalCer-related compounds have not yet yielded promising outcomes (74). Discoveries of novel NKT cell agonists (β-mannosylceramide) and improved α-GalCer analogs (α-C-GalCer) (74, 85), as well as greater understanding of tumors where this cell type is prominent may assist in harnessing the potential of NKT cells to improve T cell checkpoint therapy.

Mucosal-associated invariant T (MAIT) cells are another class of unconventional T cell that have gained much attention, given their relative abundance in humans and their association to a number of inflammatory diseases (74). MAIT cells primarily recognize a number of microbial vitamin B metabolites (such as riboflavin metabolized to 5-OP-RU) (86–89) presented by the unconventional non-polymorphic MHC I-like molecule, MR1 (90). Additional MR1-independent IL-12/18-induced activation has been reported (91, 92). Upon activation, MAIT cells are capable of producing cytokines [(IL-17, IL-2, IFN-γ, and tumor necrosis factor (TNF)], proliferate and gain cytotoxic function (93–95). In the absence of defined tumor antigens binding to tumor-derived MR1, it is reasonable to speculate that MAIT cells may be regulated by microbial antigens and may be more frequent in tumors with a microbial presence. Circulating levels of MAIT cells were reduced in patients with mucosal-originated cancers (gastric, colon, and lung), but appeared normal in patients with breast, liver, or thyroid cancer (96). In colon cancer patients, MAIT cells were shown to be preferentially enriched in the TME in comparison to unaffected tissue (96–99). Poor survival prognosis has been associated to increased levels of tumor-infiltrating MAIT cells in colon cancer patients (98). In contrast, MAIT cells did not show a correlation to patient survival in esophageal adenocarcinoma (100). In concordance with the activation of MAIT cells (TCR-MR1 or IL-12/18 cytokine), they likely elicit direct (MAIT cell to tumor cell) and indirect (MAIT cell to non-MAIT cell or IL-12/18 cytokine competition) effects, regulating host anti-tumor immunity in a TME-specific manner (74, 101). Yan et al. recently reported MR1-deficient mice (which lack MAIT cells) showed improved anti-tumor immunity when assessed using models of experimental lung metastasis, subcutaneous tumor growth, and *de novo* carcinogenesis (102). In light of these findings, a further assessment of MAIT cells in the cancer setting and their relationship to prognosis and therapeutic outcome should be determined. In addition, given a great interest in microbial modification of the TME (103–105) determining whether microbes can be used to initiate metabolic functions that promote anti-tumor immunity is also of interest.

## Limiting Immunosuppressive Factors In The TME

As well as initiation of immunosuppression by immune cell subsets, the tumor itself produces a range of molecules to enable tumor progression and facilitate immune escape (Figure 1). Many of these are soluble factors that prevent overzealous inflammation during tissue damage and infection, however also mediate tumor immune evasion. Transforming growth factor (TGF)β plays an essential role in mediating immune homeostasis, however, in the context of tumor, TGFβ has been shown to both directly promote tumor progression and initiate a broad range of immune responses. These include enhancing suppressive myeloid cell infiltrate (106, 107), disabling NK cell function, and promoting transition to group 1 innate lymphoid cells in the TME (108), as well as altering the functionality of effector T cell populations while promoting Treg immune suppression (109–111). SMAD-3, which acts downstream of TGFβ1 signaling, directly induces PD-1 expression in adoptively transferred tumor antigen-specific CD8^+^ T cells isolated from the TME (112). This suggests that PD-1-upregulation that facilitates tumor immune escape may in part be dependent on TGFβ. In addition, TGFβ is associated with excluding CD8^+^ T cell entry into the tumor core, which is known to diminish immunotherapy efficacy (113). Correspondingly, *TGF*β*1* gene expression is significantly higher in patients that show stable or progressive disease, compared to those with complete or partial responses (113). Targeting TGFβ and therapies directed toward PD-1/PD-L1 or CTLA-4 amplifies tumor control by enabling a robust T cell response with improved CD4^+^ and CD8^+^ T cell activation and CD8^+^ T cell cytokine and cell killing capacity (113–116). By inhibiting TGFβ in combination with PD-1/PD-L1 blockade, CD8^+^ T cells also display enhanced capacity to infiltrate the tumor periphery and core, promoting T cell inflammation, and resolving T cell exclusion (113, 117). This highlights that targeting TGFβ may be most effective in tumor types in which TGFβ signaling mediates immune exclusion from the TME.

Impeding the clinical utility of pan-TGFβ inhibitors (blocking isoforms TGFβ1,−2, and−3) is the potential for significant toxicity, particularly pertaining to cardiac function. Development of galunisertib (LY2157299), a TGFβRI inhibitor, has shown promise for both its anti-tumor activity and ability to modulate the TME to provide improved anti-tumor control to immunotherapies (118). However, due to toxicity concerns, intermittent administration of galunisertib has been performed in clinical trials [(119) NCT01246986]. Whether intermittent drug exposure provides selective pressure for tumor escape is unclear. As TGFβ1 appears to be the predominantly enriched in human cancers, targeting this isoform specifically may prove advantageous. Development of TGFβ1-specific inhibitors that promote synergistic anti-tumor immune responses when combined with anti-PD-1, but lack cardiovascular pathologies have been identified and may lead to greater clinical utility (120). Downstream targets of tumor-derived TGFβ activation may also be more desirable and in some cases are already being assessed for therapeutic potential alongside immune checkpoint inhibitors and other immunomodulatory compounds in the clinic.

Angiogenesis in the TME is an important component to enable nutrient accessibility and maintain tumor growth, this is driven in part by TGFβ induction of vascular endothelial growth factor (VEGF). While VEGF inhibitors have been approved for a number of indications in both oncology and vascular-related diseases, in the cancer setting interest in both anti-angiogenic and immunomodulatory properties for this target are increasing. Notably, VEGF-A promotes inhibitory checkpoint expression and transcriptional reprogramming relating to exhaustion in CD8^+^ T cells (121, 122). TOX, the transcription factor that mediates CD8^+^ T cell exhaustion, was shown to be tightly regulated by VEGF-A (122). In addition, both VEGF-A and TOX expression levels were significantly reduced in MSI-h colon cancer patients compared to patients with microsatellite stability (122). MSI-h patients have better survival outcomes in response to cancer immunotherapies, which is predominantly attributed to higher tumor mutational burden (123, 124), but may also be in part be due to reduced angiogenic factors and relieved T cell exhaustion. By combining VEGF-targeted therapies and anti-PD-1, improved anti-tumor immune response was achieved (121, 122). However, TGFβ and VEGF are not completely redundant, and co-targeting these molecules together either therapeutically or through tumor-specific genetic ablation provides additional therapeutic benefit to overcome immune tolerance in the TME (125). As clinical cohorts involving combination VEGF/TGFβ and immune checkpoint blockade treatment mature, determining which patients respond to this therapy, but also which patients are refractory and the mechanism that initiates tumor escape is of importance.

Generation of immunosuppressive adenosine limits anti-tumor immunity (126). Both CD39, which catabolizes ATP to AMP, and CD73, the enzyme that generates adenosine from ATP, are expressed by and relate to poor prognosis in a number of cancer types (127–130). Regulation of tumor-derived CD73 remains complex with multiple mediators identified, including TGFβ (131, 132). Additional evidence that CD73 expression is driven by adenosine-sensing through host-A2A adenosine receptor (A2AR) expression (133, 134), TNF (135), and hypoxia within the TME (136, 137). In melanoma, CD73 levels have been linked to low MITF expression and highly invasive tumors (135). CD73 expression appears to increase with adaptive resistance in anti-PD-1-treated melanoma patients as well as MART-1 adoptive T cell therapy (135), suggesting that tumor expression may facilitate therapeutic resistance in response to active anti-tumor immunity. In melanoma patients with innate therapeutic resistance, CD73 is not present or induced with exposure to anti-PD-1 treatment (135), likely due to a lack of inflammatory stimuli in the TME. Therapies targeting the adenosinergic pathway have been shown preclinically to potentiate the response of chemotherapies (127), immune checkpoint inhibitors (138, 139), chimeric antigen receptor T cells (140) and oncogenic BRAF inhibitors (141). This can also be through indirectly targeting the adenosine pathway, with both systemic oxygenation which relieves hypoxia-driven adenosine production, and blockade of an alternate mechanism of adenosine-production by inhibiting CD38, with both shown to potentiate the therapeutic efficacy of immune checkpoint blockade (137, 142, 143). Similarly, targeting upstream CD39 has been shown preclinically to promote therapeutic activity of immune checkpoint inhibitors (144), chemotherapies (145), and even can potentiate CD73 blockade in suboptimal concentrations (146). This highlights the complex regulatory network and the multi-faceted combination strategies involving adenosine-related molecules that may add benefit to patient care.

Targeting adenosine production and signaling have both moved forward to early phase clinical trials with promising results (NCT02403193, NCT02503774, NCT03454451). Notably, citforadenant (an A2AR inhibitor) in combination with atezolizumab (anti-PD-L1) initiated therapeutic response in both patients naïve to immunotherapies and those refractory to prior immunotherapy exposure, highlighting the potential for this combination to reinvigorate anti-tumor immunity (147). Analysis of tumor biopsies from renal cell carcinoma (RCC) preceding therapeutic intervention revealed an adenosine signature that may predict patients who will benefit from adenosine-related therapies (147). The adenosine signature was consistent with myeloid inflammation and reduced angiogenesis, both of which have been defined as poor prognostically for atezolizumab and sunitinib (tyrosine kinase inhibitor) treatment. This identifies a patient group for which adenosine may be most applicable, that inadvertently are less responsive to current clinically approved therapies for RCC. In addition, an extended disease control rate in response to citforadenant, with or without atezolizumab, was linked to improved CD8^+^ T cell infiltration (147). Adenosine has previously been shown to limit the proliferation and maturation of lymphocytic immune cell subsets (126, 134), and increased immune infiltrate in to the tumor core has been observed in response to co-targeting CD73 and A2AR in preclinical models (133). Understanding the regulation of the adenosinergic pathway in particular tumor types and in response to cancer therapies, including immunotherapy, may identify patient populations where adenosine-related therapies may be implemented with greatest success.

With increasing examination of the TME it is clear that a number of therapeutic regimens may be successfully repurposed in the treatment of cancer. For instance, targeting adenosine has been utilized previously in the setting of neurodegeneration, but has increasingly shown merit for initiating anti-tumor immunity. Additionally, aspirin may provide a combinatorial approach to overcome therapeutic resistance to immune checkpoint inhibitors. Increasing evidence demonstrates that cyclooxygenase (COX)-driven production of prostaglandins mediates anti-PD-1 resistance and limits the proinflammatory tumor milieu (148). COX enzymatic activity is disrupted by high-dose aspirin, which valuably may be repurposed to the cancer setting alongside immunotherapies to promote anti-tumor immunity. Regular aspirin users with colorectal cancer patients displaying low tumor PD-L1 expression are also afforded significantly improved survival outcomes (149). This survival advantage was not identified in PD-L1 high tumors, suggesting that engagement of the PD-1/PD-L1 axis in the TME may abrogate aspirin-mediated anti-tumor benefit and the potential utility of combination treatment. Genetic ablation of prostaglandin-endoperoxide synthase 2 (*PTSG2)*, which encodes COX-2, has been shown to promote CD8^+^ T cells and decrease the frequency of Tregs within the TME (150), both of which are predictive markers of good prognosis. Induction of COX-2 may be in part regulated by TGFβ, highlighting the complex nature of direct and indirect regulatory pathways that the tumor elicits to subdue the anti-tumor immune response. Clinical trials to develop an understanding of prostaglandin/COX-2 inhibition and immune checkpoint blockade therapeutic responses are underway in multiple tumor types [(151) NCT03396952, NCT03638297, NCT03864575, NCT03926338].

## Implementing Combination Therapeutic Regimens

**Figure 2:**
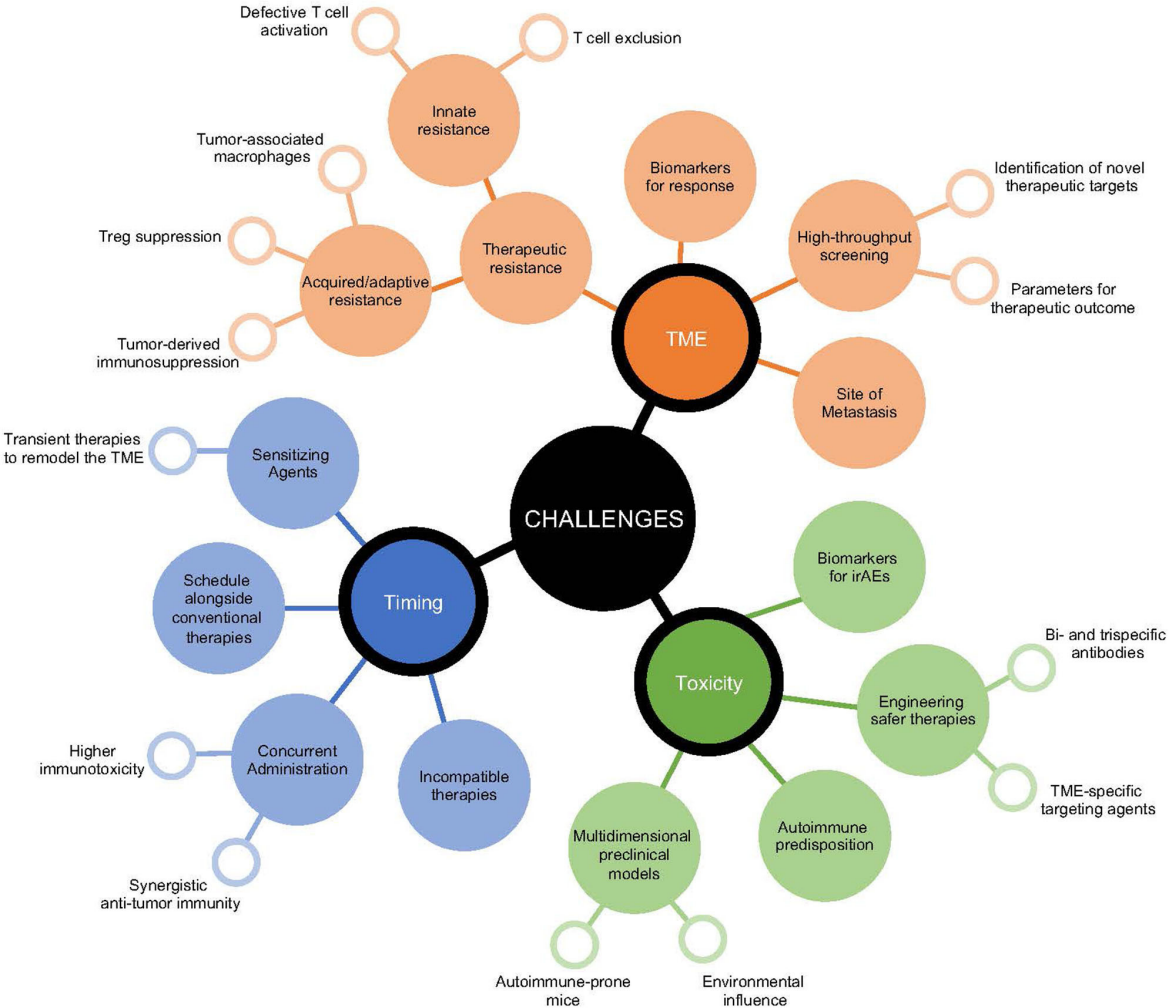
Growing challenges in the implementation of immunotherapeutic strategies. Decision-making for the most efficacious immunotherapeutic combination for cancer patients presents multiple layers of challenges. These include those surrounding the patient's tumor microenvironment, the potential risk for initiation of immunotoxicities, and the sequence for appropriate timing of therapeutic interventions. Surrounding each of these challenges are multidimensional considerations that relate to improving anti-tumor immunity and targets that influence the selection of immunomodulatory agents.

While preclinical studies have identified a number of clinically relevant therapeutic strategies to reinvigorate the immune response against cancer, their successful clinical utility has been difficult to implement (Figure 2). Combining indoleamine 2,3-dioxygenase (IDO) inhibition, an enzyme upregulated in human cancers that initiates the breakdown of tryptophan leading to multi-faceted immunosuppression within the TME (152), alongside immune checkpoint inhibitors showed promise for enhancing anti-tumor immunity in mice (153, 154). However, in a phase 3 clinical trial assessing the survival benefit for stage III/IV unresectable melanoma patients treated with epacadostat (selective IDO1 inhibitor) and pembrolizumab (anti-PD-1), this combination failed to provide additive therapeutic potential compared to pembrolizumab alone (155). Improved understanding of the TME is necessary to assist rational selection of immunotherapies required for optimal treatment outcomes. Of course, this remains a challenge even for clinically approved agents, where aside from tumor PD-L1 expression and genetic stablility of the tumor, no biomarkers for efficacy or toxicity of immune checkpoint inhibitors are approved for clinical use. Significant investment to establish biomarkers to denote responders and non-responders should be performed in early phase clinical trials to identify subgroups for which combination therapies may show greatest activity, an important first-step to facilitate response, mitigate toxicity, and minimize unnecessary cost.

In the same vein, examining optimal timing for immunotherapeutic combinations is often not well-defined. In most cases, therapeutic benefit for novel clinical agents are tested either alone or alongside concurrent anti-PD-1/PD-L1 treatment, often in cancer patients refractory to previous immune checkpoint inhibitor exposure. Preclinical evidence suggests synchronous administration of multiple immunotherapies can in some circumstances be detrimental. Two independent studies identified that concurrent administration of anti-OX40 to anti-PD-1 therapy either with or without a tumor vaccine diminished the anti-tumor immune response compared to addition of anti-OX40 alone in preclinical mouse models (156, 157). Notably, staggering the timing of these therapies where anti-OX40 preceded anti-PD-1 treatment facilitated greatest tumor control (156). Using a preclinical PDAC model, the use of gemcitabine and nab-paclitaxel, a standard chemotherapy combination for PDAC, impaired the efficacy of anti-CD40, anti-PD-1, and anti-CTLA-4 (34). Additionally, transient treatments preceding immune checkpoint inhibition can also significantly re-educate the immune response to promote anti-tumor immunity. In an anti-PD-1-resistant mouse model, a single-dose of agonistic anti-CD40 sensitized the TME to anti-PD-1 treatment in a synergistic manner (36). Strongly activating or agonistic therapies may provide greatest benefit as sensitizing agents to remodel the TME and promote entry of anti-tumor immune cells that are then targetable by immune checkpoint blockade. Alternating timing of treatments or reducing the therapeutic window for largely inflammatory combinations may also assist to potentiate therapeutic response and minimize immunotherapy-induced immune-related adverse events (irAEs).

## Challenges For Re-Educating The TME

With the advent of high-throughput screening to delineate critical components that prevent immune infiltrate or disable active anti-tumor immunity, a growing understanding of rational targets to re-ignite a therapeutic response is becoming increasingly available (158–161). This aims to equip patients who develop adaptive or acquired resistance with greater tools to re-engage the immune response against cancer and for patients with innate resistance to enable visibility of tumors (162, 163). New subgroups of cancer patients that present distinct challenges to the efficacy of immunotherapies are emerging. Of growing interest, is the relationship between metastatic site and therapeutic outcome, in which liver metastasis appears to be a major obstacle even for combination anti-PD-1 and anti-CTLA-4 treatment (164, 165). Most prominently, melanoma patients bearing liver metastasis have reduced CTLA-4 and PD-1 co-expression in CD8^+^ T cells (164), which has been shown to stratify therapeutic response to immune checkpoint inhibitor treatment (166). Efforts to provide mechanistic insight as to whether therapeutic resistance is due to myeloid cell dysfunction, Treg suppression, and immunosuppressive factors accumulating in the TME are essential, highlighting the need for tailored immunotherapies.

As the number and type of combination immunotherapies expands, the risk for increasing irAEs may also become more prevalent. Combination nivolumab (anti-PD-1) and ipilimumab (anti-CTLA-4) clearly exhibits greater levels of severe grade 3-4 immunotoxicities than either therapy alone (3, 167). Surprising levels of irAEs have been observed with other rational combinations. For instance, targeted therapies (such as BRAF inhibitors and MEK inhibitors) have been shown to potentiate immune checkpoint inhibitor activity in preclinical models (168), but when used together in melanoma patients severe irAEs were observed forcing closure of the study (169, 170). This highlights a need for improved preclinical models that emulate clinical conditions and allow for simultaneous assessment of tumor control and development of irAEs (171). Developing tumor models in autoimmune-prone mice or lowering the threshold for self-tolerance in mice with available syngeneic tumors that are resistant to autoimmune responses may facilitate improved therapeutic modeling (53, 171, 172). With the expanding use of cancer immunotherapies in more diverse populations of cancer patients, including those with pre-existing autoimmune diseases or previous immunotherapy-induced irAEs (173), or under persistent immunosuppression due to chronic viral infections (174) and allogeneic transplantation (175), developing preclinical models that incorporate multiple elements relating to tumor origin, patient history, and environment that assist in providing a more informed understanding of the clinical impact of therapeutic combinations will be essential.

As the number of immunotherapeutic targets expands, initiating smarter multi-modal strategies, to provide greater efficacy with lower toxicity will be appealing. Advancement in engineering therapies to have delayed release or greater tissue and cellular specificity have great promise. As mentioned, depletion of intratumoral Tregs without impacting peripheral Tregs would be advantageous for inducing TME-specific modulation of the CD8^+^ to Treg ratio, while avoiding toxicity induced by systemic depletion. Notably, the development of a dual variable domain anti-CTLA-4 antibody, which exhibits an outer tumor antigen-binding site that hides the CTLA-4 binding region of the antibody until reaching the TME has been shown to reduce immunotherapy-induced toxicity without impeding anti-tumor immunity (176). Similarly, bispecific or trispecific antibodies that target multiple markers upregulated in the TME on both immune cell subsets and the tumor may also lead to greater efficacy (177, 178). One such example, is the bispecific antibody combination targeting OX40 and CTLA-4, which significantly enhanced CD8^+^ T cells and reduced Tregs specifically within the TME, leading to better tumor control than either therapy alone (178). In addition to modifying the target antigen, antibodies may also be conjugated to biomaterials or nanoparticles, to ensure sustained, local release (179). Refinement made to antibodies by factoring TME properties, such as pH activation and PD-1 glycosylation (180–182), should also significantly improve the specificity and potency of immunotherapies and limit unwanted toxicity.

## Future Directions

While determining the optimal immunotherapy for an individual TME remains a challenge, encouraging is the range of available strategies to re-educate the immune response against tumor. Refinement of therapeutic targets against cellular and immunomodulatory molecules within the TME are increasing, and their promise for clinical utility is growing. Importantly, greater effort in defining the therapeutic setting where each may be applicable will be essential for clinical success. Since anti-PD-1/PD-L1 has shown value in multiple modalities, examining tumor types where anti-PD-1 activity is limited may yield greatest therapeutic breakthroughs in identifying strategies to remodel inert immune circumstances in the tumor. In deciding on the use of these therapies, a cost-benefit analysis relating to the purported immunotoxicity and likelihood for the immunotherapy strategy to enhance anti-tumor immunity is necessary. This emphasizes a need for rational and selective combination immunotherapies to be utilized within a defined TME in order to re-educate the anti-tumor immune response.

## Author Contributions

SN and AY contributed equally to the writing and revision of the manuscript. All authors contributed to the article and approved the submitted version.

## Conflict of Interest

The authors declare that the research was conducted in the absence of any commercial or financial relationships that could be construed as a potential conflict of interest.

## Abbreviations

A2AR: A2A adenosine receptor
APCs: antigen presenting cells
CSF1: colony stimulating factor 1
CSF1R: colony stimulating factor 1 receptor
COX: cyclooxygenase
CTLA-4: cytotoxic T-lymphocyte associated protein 4
dMMR: deficient DNA mismatch repair
EZH2: enhancer of zeste homolog 2
FLT3L: FMS-like tyrosine kinase 3 ligand
IDO: indoleamine 2,3-dioxygenase
IFN: interferon
irAEs: immune-related adverse events
IL: interleukin
MAIT: mucosal-associated invariant T
MSI-h: microsatellite instability-hi
NKT: natural killer T
NRP1: neuropilin-1
NSCLC: non-small cell lung cancer
PDAC: pancreatic ductal adenocarcinoma
PD-1: programmed death-1
PD-L1: programmed death-ligand 1
PMN-MDSC: polymorphonuclear myeloid-derived suppressor cells
PTSG2: prostaglandin-endoperoxide synthase 2
RCC: renal cell carcinoma
Tregs: regulatory T cells
TAMs: tumor-associated macrophages
TGF: transforming growth factor
TILs: tumor-infiltrating lymphocytes
TME: tumor microenvironment
TNF: tumor necrosis factor
VEGF: vascular endothelial growth factor.

## References

[1] Khalil DN Smith EL Brentjens RJ Wolchok JD The future of cancer treatment: immunomodulation, CARs and combination immunotherapy. Nat Rev Clin Oncol. (2016) 13:394 10.1038/nrclinonc.2016.65 27118494 PMC5558237

[2] Hammers HJ Plimack ER Infante JR Rini BI McDermott DF Lewis LD. Safety and efficacy of nivolumab in combination with ipilimumab in metastatic renal cell carcinoma: the checkmate 016 study. J Clin Oncol. (2017) 35:3851–8. 10.1200/JCO.2016.72.1985 28678668 PMC7587408

[3] Wolchok JD Chiarion-Sileni V Gonzalez R Rutkowski P Grob JJ Cowey CL. Overall survival with combined nivolumab and ipilimumab in advanced melanoma. N Engl J Med. (2017) 377:1345–56. 10.1056/NEJMoa1709684 28889792 PMC5706778

[4] Rizvi NA Hellmann MD Snyder A Kvistborg P Makarov V Havel JJ. Cancer immunology. mutational landscape determines sensitivity to PD-1 blockade in non-small cell lung cancer. Science. (2015) 348:124–8. 10.1126/science.aaa1348 25765070 PMC4993154

[5] Goodman AM Kato S Bazhenova L Patel SP Frampton GM Miller V. Tumor mutational burden as an independent predictor of response to immunotherapy in diverse cancers. Mol Cancer Ther. (2017) 16:2598–608. 10.1158/1535-7163.MCT-17-0386 28835386 PMC5670009

[6] Yarchoan M Hopkins A Jaffee EM. Tumor mutational burden and response rate to PD-1 inhibition. N Engl J Med. (2017) 377:2500–1. 10.1056/NEJMc1713444 29262275 PMC6549688

[7] Teng MW Ngiow SF Ribas A Smyth MJ. Classifying cancers based on T-cell infiltration and PD-L1. Cancer Res. (2015) 75:2139–45. 10.1158/0008-5472.CAN-15-0255 25977340 PMC4452411

[8] Twyman-Saint Victor C Rech AJ Maity A Rengan R Pauken KE Stelekati E. Radiation and dual checkpoint blockade activate non-redundant immune mechanisms in cancer. Nature. (2015) 520:373–7. 10.1038/nature14292 25754329 PMC4401634

[9] Wei SC Levine JH Cogdill AP Zhao Y Anang NAS Andrews MC. Distinct cellular mechanisms underlie anti-CTLA-4 and anti-PD-1 checkpoint blockade. Cell. (2017) 170:1120–33 e17. 10.1016/j.cell.2017.07.024 28803728 PMC5591072

[10] Uno T Takeda K Kojima Y Yoshizawa H Akiba H Mittler RS. Eradication of established tumors in mice by a combination antibody-based therapy. Nat Med. (2006) 12:693–8. 10.1038/nm1405 16680149

[11] Moynihan KD Opel CF Szeto GL Tzeng A Zhu EF Engreitz JM. Eradication of large established tumors in mice by combination immunotherapy that engages innate and adaptive immune responses. Nat Med. (2016) 22:1402–10. 10.1038/nm.4200 27775706 PMC5209798

[12] Franklin RA Liao W Sarkar A Kim MV Bivona MR Liu K. The cellular and molecular origin of tumor-associated macrophages. Science. (2014) 344:921–5. 10.1126/science.1252510 24812208 PMC4204732

[13] Zhu Y Herndon JM Sojka DK Kim KW Knolhoff BL Zuo C Tissue-resident macrophages in pancreatic ductal adenocarcinoma originate from embryonic hematopoiesis and promote tumor progression. Immunity. (2017) 47:323–38.e6. 10.1016/j.immuni.2017.07.014 28813661 PMC5578409

[14] Gubin MM Esaulova E Ward JP Malkova ON Runci D Wong P High-dimensional analysis delineates myeloid and lymphoid compartment remodeling during successful immune-checkpoint cancer therapy. Cell. (2018) 175:1014–30.e19. 10.1016/j.cell.2018.09.030 30343900 PMC6501221

[15] DeNardo DG Andreu P Coussens LM. Interactions between lymphocytes and myeloid cells regulate pro- versus anti-tumor immunity. Cancer Metastasis Rev. (2010) 29:309–16. 10.1007/s10555-010-9223-6 20405169 PMC2865635

[16] Veglia F Perego M Gabrilovich D. Myeloid-derived suppressor cells coming of age. Nat Immunol. (2018) 19:108–19. 10.1038/s41590-017-0022-x 29348500 PMC5854158

[17] Arlauckas SP Garris CS Kohler RH Kitaoka M Cuccarese MF Yang KS. *In vivo* imaging reveals a tumor-associated macrophage-mediated resistance pathway in anti-PD-1 therapy. Sci Transl Med. (2017) 9:eaal3604. 10.1126/scitranslmed.aal3604 28490665 PMC5734617

[18] Dahan R Sega E Engelhardt J Selby M Korman AJ Ravetch JV FcgammaRs modulate the anti-tumor activity of antibodies targeting the PD-1/PD-L1 axis. Cancer Cell. (2015) 28:285–95. 10.1016/j.ccell.2015.08.004 26373277

[19] Wynn TA Chawla A Pollard JW. Macrophage biology in development, homeostasis and disease. Nature. (2013) 496:445–55. 10.1038/nature12034 23619691 PMC3725458

[20] Strachan DC Ruffell B Oei Y Bissell MJ Coussens LM Pryer N. CSF1R inhibition delays cervical and mammary tumor growth in murine models by attenuating the turnover of tumor-associated macrophages and enhancing infiltration by CD8(+) T cells. Oncoimmunology. (2013) 2:e26968. 10.4161/onci.26968 24498562 PMC3902121

[21] Zhu Y Knolhoff BL Meyer MA Nywening TM West BL Luo J. CSF1/CSF1R blockade reprograms tumor-infiltrating macrophages and improves response to T-cell checkpoint immunotherapy in pancreatic cancer models. Cancer Res. (2014) 74:5057–69. 10.1158/0008-5472.CAN-13-3723 25082815 PMC4182950

[22] Mok S Koya RC Tsui C Xu J Robert L Wu L. Inhibition of CSF-1 receptor improves the antitumor efficacy of adoptive cell transfer immunotherapy. Cancer Res. (2014) 74:153–61. 10.1158/0008-5472.CAN-13-1816 24247719 PMC3947337

[23] Ngiow SF Meeth KM Stannard K Barkauskas DS Bollag G Bosenberg M. Co-inhibition of colony stimulating factor-1 receptor and BRAF oncogene in mouse models of BRAF(V600E) melanoma. Oncoimmunology. (2016) 5:e1089381. 10.1080/2162402X.2015.1089381 27141346 PMC4839378

[24] DeNardo DG Brennan DJ Rexhepaj E Ruffell B Shiao SL Madden SF. Leukocyte complexity predicts breast cancer survival and functionally regulates response to chemotherapy. Cancer Discov. (2011) 1:54–67. 10.1158/2159-8274.CD-10-0028 22039576 PMC3203524

[25] Kumar V Donthireddy L Marvel D Condamine T Wang F Lavilla-Alonso S. Cancer-associated fibroblasts neutralize the anti-tumor effect of CSF1 receptor blockade by inducing PMN-MDSC infiltration of tumors. Cancer Cell. (2017) 32:654–68.e5. 10.1016/j.ccell.2017.10.005 29136508 PMC5827952

[26] Teng KY Han J Zhang X Hsu SH He S Wani NA. Blocking the CCL2-CCR2 axis using CCL2-neutralizing antibody is an effective therapy for hepatocellular cancer in a mouse model. Mol Cancer Ther. (2017) 16:312–22. 10.1158/1535-7163.MCT-16-0124 27980102 PMC5292068

[27] Mitchem JB Brennan DJ Knolhoff BL Belt BA Zhu Y Sanford DE. Targeting tumor-infiltrating macrophages decreases tumor-initiating cells, relieves immunosuppression, and improves chemotherapeutic responses. Cancer Res. (2013) 73:1128–41. 10.1158/0008-5472.CAN-12-2731 23221383 PMC3563931

[28] Wu X Singh R Hsu DK Zhou Y Yu S Han D A small molecule CCR2 antagonist depletes tumor macrophages and synergizes with anti-PD-1 in a murine model of cutaneous T-cell lymphoma (CTCL). J Invest Dermatol. (2020) 140:1390–400.e4. 10.1016/j.jid.2019.11.018 31945344

[29] Grossman JG Nywening TM Belt BA Panni RZ Krasnick BA DeNardo DG. Recruitment of CCR2(+) tumor associated macrophage to sites of liver metastasis confers a poor prognosis in human colorectal cancer. Oncoimmunology. (2018) 7:e1470729. 10.1080/2162402X.2018.1470729 30228938 PMC6140580

[30] House IG Savas P Lai J Chen AXY Oliver AJ Teo ZL. Macrophage-derived CXCL9 and CXCL10 are required for antitumor immune responses following immune checkpoint blockade. Clin Cancer Res. (2020) 26:487–504. 10.1158/1078-0432.CCR-19-1868 31636098

[31] Clark CE Hingorani SR Mick R Combs C Tuveson DA Vonderheide RH. Dynamics of the immune reaction to pancreatic cancer from inception to invasion. Cancer Res. (2007) 67:9518–27. 10.1158/0008-5472.CAN-07-0175 17909062

[32] Aiello NM Bajor DL Norgard RJ Sahmoud A Bhagwat N Pham MN. Metastatic progression is associated with dynamic changes in the local microenvironment. Nat Commun. (2016) 7:12819. 10.1038/ncomms12819 27628423 PMC5027614

[33] Vonderheide RH. The immune revolution: a case for priming, not checkpoint. Cancer Cell. (2018) 33:563–9. 10.1016/j.ccell.2018.03.008 29634944 PMC5898647

[34] Morrison AH Diamond MS Hay CA Byrne KT Vonderheide RH. Sufficiency of CD40 activation and immune checkpoint blockade for T cell priming and tumor immunity. Proc Natl Acad Sci USA. (2020) 117:8022–31. 10.1073/pnas.1918971117 32213589 PMC7149500

[35] Luheshi NM Coates-Ulrichsen J Harper J Mullins S Sulikowski MG Martin P. Transformation of the tumour microenvironment by a CD40 agonist antibody correlates with improved responses to PD-L1 blockade in a mouse orthotopic pancreatic tumour model. Oncotarget. (2016) 7:18508–20. 10.18632/oncotarget.7610 26918344 PMC4951305

[36] Ngiow SF Young A Blake SJ Hill GR Yagita H Teng MW. Agonistic CD40 mAb-driven IL12 reverses resistance to anti-PD1 in a T-cell-rich tumor. Cancer Res. (2016) 76:6266–77. 10.1158/0008-5472.CAN-16-2141 27634762

[37] Salmon H Idoyaga J Rahman A Leboeuf M Remark R Jordan S. Expansion and activation of CD103(+) dendritic cell progenitors at the tumor site enhances tumor responses to therapeutic PD-L1 and BRAF inhibition. Immunity. (2016) 44:924–38. 10.1016/j.immuni.2016.03.012 27096321 PMC4980762

[38] Hildner K Edelson BT Purtha WE Diamond M Matsushita H Kohyama M. Batf3 deficiency reveals a critical role for CD8alpha+ dendritic cells in cytotoxic T cell immunity. Science. (2008) 322:1097–100. 10.1126/science.1164206 19008445 PMC2756611

[39] Broz ML Binnewies M Boldajipour B Nelson AE Pollack JL Erle DJ Dissecting the tumor myeloid compartment reveals rare activating antigen-presenting cells critical for T cell immunity. Cancer Cell. (2014) 26:638–52. 10.1016/j.ccell.2014.09.007 25446897 PMC4254577

[40] Ruffell B Chang-Strachan D Chan V Rosenbusch A Ho CM Pryer N. Macrophage IL-10 blocks CD8+ T cell-dependent responses to chemotherapy by suppressing IL-12 expression in intratumoral dendritic cells. Cancer Cell. (2014) 26:623–37. 10.1016/j.ccell.2014.09.006 25446896 PMC4254570

[41] Roberts EW Broz ML Binnewies M Headley MB Nelson AE Wolf DM. Critical role for CD103(+)/CD141(+) dendritic cells bearing CCR7 for tumor antigen trafficking and priming of T cell immunity in melanoma. Cancer Cell. (2016) 30:324–36. 10.1016/j.ccell.2016.06.003 27424807 PMC5374862

[42] Beavis PA Henderson MA Giuffrida L Davenport AJ Petley EV House IG. Dual PD-1 and CTLA-4 checkpoint blockade promotes antitumor immune responses through CD4(+)Foxp3(-) cell-mediated modulation of CD103(+) dendritic cells. Cancer Immunol Res. (2018) 6:1069–81. 10.1158/2326-6066.CIR-18-0291 30018045

[43] Barry KC Hsu J Broz ML Cueto FJ Binnewies M Combes AJ. A natural killer-dendritic cell axis defines checkpoint therapy-responsive tumor microenvironments. Nat Med. (2018) 24:1178–91. 10.1038/s41591-018-0085-8 29942093 PMC6475503

[44] Maier B Chen ST Tung N Chang C LeBerichel J Chudnovskiy A A conserved dendritic-cell regulatory program limits antitumour immunity. Nature. (2020) 508:257–62. 10.1038/s41586-020-2134-y PMC7787191 32269339

[45] Fridman WH Pages F Sautes-Fridman C Galon J. The immune contexture in human tumours: impact on clinical outcome. Nat Rev Cancer. (2012) 12:298–306. 10.1038/nrc3245 22419253

[46] Shang B Liu Y Jiang SJ Liu Y. Prognostic value of tumor-infiltrating FoxP3+ regulatory T cells in cancers: a systematic review and meta-analysis. Sci Rep. (2015) 5:15179. 10.1038/srep15179 26462617 PMC4604472

[47] Tanaka A Sakaguchi S Regulatory T cells in cancer immunotherapy. Cell Res. (2017) 27:109–18. 10.1038/cr.2016.151 27995907 PMC5223231

[48] Binnewies M Mujal AM Pollack JL Combes AJ Hardison EA Barry KC. Unleashing type-2 dendritic cells to drive protective antitumor CD4(+) T cell immunity. Cell. (2019) 177:556–71e16. 10.1016/j.cell.2019.02.005 30955881 PMC6954108

[49] Qi S Li H Lu L Qi Z Liu L Chen L. Long-term intravital imaging of the multicolor-coded tumor microenvironment during combination immunotherapy. Elife. (2016) 5:e14756. 10.7554/eLife.14756 27855783 PMC5173323

[50] Taylor NA Vick SC Iglesia MD Brickey WJ Midkiff BR McKinnon KP. Treg depletion potentiates checkpoint inhibition in claudin-low breast cancer. J Clin Invest. (2017) 127:3472–83. 10.1172/JCI90499 28825599 PMC5669567

[51] Sakuishi K Ngiow SF Sullivan JM Teng MW Kuchroo VK Smyth MJ. TIM3(+)FOXP3(+) regulatory T cells are tissue-specific promoters of T-cell dysfunction in cancer. Oncoimmunology. (2013) 2:e23849. 10.4161/onci.23849 23734331 PMC3654601

[52] Penaloza-MacMaster P Kamphorst AO Wieland A Araki K Iyer SS West EE. Interplay between regulatory T cells and PD-1 in modulating T cell exhaustion and viral control during chronic LCMV infection. J Exp Med. (2014) 211:1905–18. 10.1084/jem.20132577 25113973 PMC4144726

[53] Liu J Blake SJ Harjunpaa H Fairfax KA Yong MC Allen S. Assessing immune-related adverse events of efficacious combination immunotherapies in preclinical models of cancer. Cancer Res. (2016) 76:5288–301. 10.1158/0008-5472.CAN-16-0194 27503925

[54] Coe D Begom S Addey C White M Dyson J Chai JG. Depletion of regulatory T cells by anti-GITR mAb as a novel mechanism for cancer immunotherapy. Cancer Immunol Immunother. (2010) 59:1367–77. 10.1007/s00262-010-0866-5 20480365 PMC11030908

[55] Simpson TR Li F Montalvo-Ortiz W Sepulveda MA Bergerhoff K Arce F. Fc-dependent depletion of tumor-infiltrating regulatory T cells co-defines the efficacy of anti-CTLA-4 therapy against melanoma. J Exp Med. (2013) 210:1695–710. 10.1084/jem.20130579 23897981 PMC3754863

[56] Selby MJ Engelhardt JJ Quigley M Henning KA Chen T Srinivasan M. Anti-CTLA-4 antibodies of IgG2a isotype enhance antitumor activity through reduction of intratumoral regulatory T cells. Cancer Immunol Res. (2013) 1:32–42. 10.1158/2326-6066.CIR-13-0013 24777248

[57] Bulliard Y Jolicoeur R Zhang J Dranoff G Wilson NS Brogdon JL. OX40 engagement depletes intratumoral tregs via activating FcγRs, leading to antitumor efficacy. Immunol Cell Biol. (2014) 92:475–80. 10.1038/icb.2014.26 24732076

[58] Arce Vargas F Furness AJS Litchfield K Joshi K Rosenthal R Ghorani E. Fc effector function contributes to the activity of human anti-CTLA-4 antibodies. Cancer Cell. (2018) 33:649–63.e4. 10.1016/j.ccell.2018.02.010 29576375 PMC5904288

[59] Ingram JR Blomberg OS Rashidian M Ali L Garforth S Fedorov E. Anti-CTLA-4 therapy requires an Fc domain for efficacy. Proc Natl Acad Sci USA. (2018) 115:3912–7. 10.1073/pnas.1801524115 29581255 PMC5899492

[60] Sugiyama D Nishikawa H Maeda Y Nishioka M Tanemura A Katayama I. Anti-CCR4 mAb selectively depletes effector-type FoxP3+CD4+ regulatory T cells, evoking antitumor immune responses in humans. Proc Natl Acad Sci USA. (2013) 110:17945–50. 10.1073/pnas.1316796110 24127572 PMC3816454

[61] Doi T Muro K Ishii H Kato T Tsushima T Takenoyama M A phase I study of the anti-CC chemokine receptor 4 antibody, mogamulizumab, in combination with nivolumab in patients with advanced or metastatic solid tumors. Clin Cancer Res. (2019) 25:6614–22. 10.1158/1078-0432.CCR-19-1090 31455681

[62] Kurtulus S Sakuishi K Ngiow SF Joller N Tan DJ Teng MW. TIGIT predominantly regulates the immune response via regulatory T cells. J Clin Invest. (2015) 125:4053–62. 10.1172/JCI81187 26413872 PMC4639980

[63] Bournazos S Wang TT Dahan R Maamary J Ravetch JV. Signaling by antibodies: recent progress. Annu Rev Immunol. (2017) 35:285–311. 10.1146/annurev-immunol-051116-052433 28446061 PMC5613280

[64] Arce Vargas F Furness AJS Solomon I Joshi K Mekkaoui L Lesko MH. Fc-optimized anti-CD25 depletes tumor-infiltrating regulatory T cells and synergizes with PD-1 blockade to eradicate established tumors. Immunity. (2017) 46:577–86. 10.1016/j.immuni.2017.03.013 28410988 PMC5437702

[65] Josefowicz SZ Lu LF Rudensky AY. Regulatory T cells: mechanisms of differentiation and function. Annu Rev Immunol. (2012) 30:531–64. 10.1146/annurev.immunol.25.022106.141623 22224781 PMC6066374

[66] van Gool F Nguyen MLT Mumbach MR Satpathy AT Rosenthal WL Giacometti S. A mutation in the transcription factor Foxp3 drives T helper 2 effector function in regulatory T cells. Immunity. (2019) 50:362–77.e6. 10.1016/j.immuni.2018.12.016 30709738 PMC6476426

[67] Francisco LM Sage PT Sharpe AH. The PD-1 pathway in tolerance and autoimmunity. Immunol Rev. (2010) 236:219–42. 10.1111/j.1600-065X.2010.00923.x 20636820 PMC2919275

[68] Wan YY Flavell RA. Regulatory T-cell functions are subverted and converted owing to attenuated Foxp3 expression. Nature. (2007) 445:766–70. 10.1038/nature05479 17220876

[69] Delgoffe GM Woo SR Turnis ME Gravano DM Guy C Overacre AE. Stability and function of regulatory T cells is maintained by a neuropilin-1-semaphorin-4a axis. Nature. (2013) 501:252–6. 10.1038/nature12428 23913274 PMC3867145

[70] Overacre-Delgoffe AE Chikina M Dadey RE Yano H Brunazzi EA Shayan G. Interferon-gamma drives Treg fragility to promote anti-tumor immunity. Cell. (2017) 169:1130–41.e11. 10.1016/j.cell.2017.05.005 28552348 PMC5509332

[71] DuPage M Chopra G Quiros J Rosenthal WL Morar MM Holohan D. The chromatin-modifying enzyme Ezh2 is critical for the maintenance of regulatory T cell identity after activation. Immunity. (2015) 42:227–38. 10.1016/j.immuni.2015.01.007 25680271 PMC4347854

[72] Wang D Quiros J Mahuron K Pai CC Ranzani V Young A. Targeting EZH2 reprograms intratumoral regulatory T cells to enhance cancer immunity. Cell Rep. (2018) 23:3262–74. 10.1016/j.celrep.2018.05.050 29898397 PMC6094952

[73] Goswami S Apostolou I Zhang J Skepner J Anandhan S Zhang X. Modulation of EZH2 expression in T cells improves efficacy of anti-CTLA-4 therapy. J Clin Invest. (2018) 128:3813–8. 10.1172/JCI99760 29905573 PMC6118570

[74] Godfrey DI Le Nours J Andrews DM Uldrich AP Rossjohn J. Unconventional T cell targets for cancer immunotherapy. Immunity. (2018) 48:453–73. 10.1016/j.immuni.2018.03.009 29562195

[75] Kawano T Cui J Koezuka Y Toura I Kaneko Y Motoki K. CD1d-restricted and TCR-mediated activation of valpha14 NKT cells by glycosylceramides. Science. (1997) 278:1626–9. 10.1126/science.278.5343.1626 9374463

[76] Metelitsa LS Naidenko OV Kant A Wu HW Loza MJ Perussia B Human NKT cells mediate antitumor cytotoxicity directly by recognizing target cell CD1d with bound ligand or indirectly by producing IL-2 to activate NK cells. J Immunol. (2001) 167:3114–22. 10.4049/jimmunol.167.6.3114 11544296

[77] Coquet JM Chakravarti S Kyparissoudis K McNab FW Pitt LA McKenzie BS. Diverse cytokine production by NKT cell subsets and identification of an IL-17-producing CD4-NK1.1- NKT cell population. Proc Natl Acad Sci USA. (2008) 105:11287–92. 10.1073/pnas.0801631105 18685112 PMC2516267

[78] McEwen-Smith RM Salio M Cerundolo V. The regulatory role of invariant NKT cells in tumor immunity. Cancer Immunol Res. (2015) 3:425–35. 10.1158/2326-6066.CIR-15-0062 25941354 PMC4430818

[79] Hermans IF Silk JD Gileadi U Salio M Mathew B Ritter G. NKT cells enhance CD4+ and CD8+ T cell responses to soluble antigen *in vivo* through direct interaction with dendritic cells. J Immunol. (2003) 171:5140–7. 10.4049/jimmunol.171.10.5140 14607913

[80] Fujii S Shimizu K Smith C Bonifaz L Steinman RM. Activation of natural killer T cells by alpha-galactosylceramide rapidly induces the full maturation of dendritic cells *in vivo* and thereby acts as an adjuvant for combined CD4 and CD8 T cell immunity to a coadministered protein. J Exp Med. (2003) 198:267–79. 10.1084/jem.20030324 12874260 PMC2194082

[81] Silk JD Hermans IF Gileadi U Chong TW Shepherd D Salio M. Utilizing the adjuvant properties of CD1d-dependent NK T cells in T cell-mediated immunotherapy. J Clin Invest. (2004) 114:1800–11. 10.1172/JCI200422046 15599405 PMC535067

[82] Song L Asgharzadeh S Salo J Engell K Wu HW Sposto R. Valpha24-invariant NKT cells mediate antitumor activity via killing of tumor-associated macrophages. J Clin Invest. (2009) 119:1524–36. 10.1172/JCI37869 19411762 PMC2689106

[83] Bae EA Seo H Kim BS Choi J Jeon I Shin KS. Activation of NKT cells in an anti-PD-1-resistant tumor model enhances antitumor immunity by reinvigorating exhausted CD8 T cells. Cancer Res. (2018) 78:5315–26. 10.1158/0008-5472.CAN-18-0734 30012672

[84] Ishii K Shimizu M Kogo H Negishi Y Tamura H Morita R. A combination of check-point blockade and alpha-galactosylceramide elicits long-lasting suppressive effects on murine hepatoma cell growth *in vivo*. Immunobiology. (2020) 225:151860. 10.1016/j.imbio.2019.10.009 31812347

[85] O'Konek JJ Illarionov P Khursigara DS Ambrosino E Izhak L Castillo BF. Mouse and human iNKT cell agonist β-mannosylceramide reveals a distinct mechanism of tumor immunity. J Clin Invest. (2011) 121:683–94. 10.1172/JCI42314 21245578 PMC3026717

[86] Awad W Le Nours J Kjer-Nielsen L McCluskey J Rossjohn J. Mucosal-associated invariant T cell receptor recognition of small molecules presented by MR1. Immunol Cell Biol. (2018) 96:588–97. 10.1111/imcb.12017 29393543

[87] Corbett AJ Eckle SB Birkinshaw RW Liu L Patel O Mahony J. T-cell activation by transitory neo-antigens derived from distinct microbial pathways. Nature. (2014) 509:361–5. 10.1038/nature13160 24695216

[88] Kjer-Nielsen L Patel O Corbett AJ Le Nours J Meehan B Liu L. MR1 presents microbial vitamin B metabolites to MAIT cells. Nature. (2012) 491:717–23. 10.1038/nature11605 23051753

[89] Mascanfroni ID Yeste A Vieira SM Burns EJ Patel B Sloma I. IL-27 acts on DCs to suppress the T cell response and autoimmunity by inducing expression of the immunoregulatory molecule CD39. Nat Immunol. (2013) 14:1054–63. 10.1038/ni.2695 23995234 PMC3964005

[90] Treiner E Duban L Bahram S Radosavljevic M Wanner V Tilloy F. Selection of evolutionarily conserved mucosal-associated invariant T cells by MR1. Nature. (2003) 422:164–9. 10.1038/nature01433 12634786

[91] Loh L Wang Z Sant S Koutsakos M Jegaskanda S Corbett AJ. Human mucosal-associated invariant T cells contribute to antiviral influenza immunity via IL-18-dependent activation. Proc Natl Acad Sci USA. (2016) 113:10133–8. 10.1073/pnas.1610750113 27543331 PMC5018778

[92] van Wilgenburg B Scherwitzl I Hutchinson EC Leng T Kurioka A Kulicke C. MAIT cells are activated during human viral infections. Nat Commun. (2016) 7:11653. 10.1038/ncomms11653 27337592 PMC4931007

[93] Gherardin NA Souter MN Koay HF Mangas KM Seemann T Stinear TP. Human blood MAIT cell subsets defined using MR1 tetramers. Immunol Cell Biol. (2018) 96:507–25. 10.1111/imcb.12021 29437263 PMC6446826

[94] Le Bourhis L Dusseaux M Bohineust A Bessoles S Martin E Premel V. MAIT cells detect and efficiently lyse bacterially-infected epithelial cells. PLoS Pathog. (2013) 9:e1003681. 10.1371/journal.ppat.1003681 24130485 PMC3795036

[95] Kurioka A Ussher JE Cosgrove C Clough C Fergusson JR Smith K. MAIT cells are licensed through granzyme exchange to kill bacterially sensitized targets. Mucosal Immunol. (2015) 8:429–40. 10.1038/mi.2014.81 25269706 PMC4288950

[96] Won EJ Ju JK Cho YN Jin HM Park KJ Kim TJ. Clinical relevance of circulating mucosal-associated invariant T cell levels and their anti-cancer activity in patients with mucosal-associated cancer. Oncotarget. (2016) 7:76274–90. 10.18632/oncotarget.11187 27517754 PMC5342813

[97] Sundstrom P Ahlmanner F Akeus P Sundquist M Alsen S Yrlid U. Human mucosa-associated invariant T cells accumulate in colon adenocarcinomas but produce reduced amounts of IFN-gamma. J Immunol. (2015) 195:3472–81. 10.4049/jimmunol.1500258 26297765

[98] Zabijak L Attencourt C Guignant C Chatelain D Marcelo P Marolleau JP. Increased tumor infiltration by mucosal-associated invariant T cells correlates with poor survival in colorectal cancer patients. Cancer Immunol Immunother. (2015) 64:1601–8. 10.1007/s00262-015-1764-7 26497850 PMC11028701

[99] Ling L Lin Y Zheng W Hong S Tang X Zhao P. Circulating and tumor-infiltrating mucosal associated invariant T (MAIT) cells in colorectal cancer patients. Sci Rep. (2016) 6:20358. 10.1038/srep20358 26837580 PMC4738248

[100] Melo AM O'Brien AM Phelan JJ Kennedy SA Wood NAW Veerapen N. Mucosal-associated invariant T cells display diminished effector capacity in oesophageal adenocarcinoma. Front Immunol. (2019) 10:1580. 10.3389/fimmu.2019.01580 31354725 PMC6635552

[101] Godfrey DI Koay HF McCluskey J Gherardin NA. The biology and functional importance of MAIT cells. Nat Immunol. (2019) 20:1110–28. 10.1038/s41590-019-0444-8 31406380

[102] Yan J Allen S McDonald E Das I Mak JYW Liu L. MAIT cells promote tumor initiation, growth, and metastases via tumor MR1. Cancer Discov. (2020) 10:124–41. 10.1158/2159-8290.CD-19-0569 31826876

[103] Gopalakrishnan V Spencer CN Nezi L Reuben A Andrews MC Karpinets TV. Gut microbiome modulates response to anti-PD-1 immunotherapy in melanoma patients. Science. (2018) 359:97–103. 10.1126/science.aan4236 29097493 PMC5827966

[104] Vetizou M Pitt JM Daillere R Lepage P Waldschmitt N Flament C. Anticancer immunotherapy by CTLA-4 blockade relies on the gut microbiota. Science. (2015) 350:1079–84. 10.1126/science.aad1329 26541610 PMC4721659

[105] Sivan A Corrales L Hubert N Williams JB Aquino-Michaels K Earley ZM. Commensal Bifidobacterium promotes antitumor immunity and facilitates anti-PD-L1 efficacy. Science. (2015) 350:1084–9. 10.1126/science.aac4255 26541606 PMC4873287

[106] Pang Y Gara SK Achyut BR Li Z Yan HH Day CP. TGF-β signaling in myeloid cells is required for tumor metastasis. Cancer Discov. (2013) 3:936–51. 10.1158/2159-8290.CD-12-0527 23661553 PMC4678771

[107] Novitskiy SV Pickup MW Chytil A Polosukhina D Owens P Moses HL. Deletion of TGF-β signaling in myeloid cells enhances their anti-tumorigenic properties. J Leukoc Biol. (2012) 92:641–51. 10.1189/jlb.1211639 22685318 PMC3427612

[108] Gao Y Souza-Fonseca-Guimaraes F Bald T Ng SS Young A Ngiow SF. Tumor immunoevasion by the conversion of effector NK cells into type 1 innate lymphoid cells. Nat Immunol. (2017) 18:1004–15. 10.1038/ni.3800 28759001

[109] Thomas DA Massague J. TGF-β directly targets cytotoxic T cell functions during tumor evasion of immune surveillance. Cancer Cell. (2005) 8:369–80. 10.1016/j.ccr.2005.10.012 16286245

[110] Chen ML Pittet MJ Gorelik L Flavell RA Weissleder R von Boehmer H. Regulatory T cells suppress tumor-specific CD8 T cell cytotoxicity through TGF-β signals *in vivo*. Proc Natl Acad Sci USA. (2005) 102:419–24. 10.1073/pnas.0408197102 15623559 PMC544311

[111] Budhu S Schaer DA Li Y Toledo-Crow R Panageas K Yang X. Blockade of surface-bound TGF-β on regulatory T cells abrogates suppression of effector T cell function in the tumor microenvironment. Sci Signal. (2017) 10:eaak9702. 10.1126/scisignal.aak9702 28851824 PMC5851440

[112] Park BV Freeman ZT Ghasemzadeh A Chattergoon MA Rutebemberwa A Steigner J. TGFβ1-mediated SMAD3 enhances PD-1 expression on antigen-specific T cells in cancer. Cancer Discov. (2016) 6:1366–81. 10.1158/2159-8290.CD-15-1347 27683557 PMC5295786

[113] Mariathasan S Turley SJ Nickles D Castiglioni A Yuen K Wang Y. TGFβ attenuates tumour response to PD-L1 blockade by contributing to exclusion of T cells. Nature. (2018) 554:544–8. 10.1038/nature25501 29443960 PMC6028240

[114] Sow HS Ren J Camps M Ossendorp F Ten Dijke P. Combined inhibition of TGF-β signaling and the PD-L1 immune checkpoint is differentially effective in tumor models. Cells. (2019) 8:320. 10.3390/cells8040320 30959852 PMC6523576

[115] Principe DR Park A Dorman MJ Kumar S Viswakarma N Rubin J. TGFβ blockade augments PD-1 inhibition to promote T-cell-mediated regression of pancreatic cancer. Mol Cancer Ther. (2019) 18:613–20. 10.1158/1535-7163.MCT-18-0850 30587556 PMC6397698

[116] Ravi R Noonan KA Pham V Bedi R Zhavoronkov A Ozerov IV. Bifunctional immune checkpoint-targeted antibody-ligand traps that simultaneously disable TGFβ enhance the efficacy of cancer immunotherapy. Nat Commun. (2018) 9:741. 10.1038/s41467-017-02696-6 29467463 PMC5821872

[117] Dodagatta-Marri E Meyer DS Reeves MQ Paniagua R To MD Binnewies M. α-PD-1 therapy elevates Treg/Th balance and increases tumor cell pSmad3 that are both targeted by alpha-TGFβ antibody to promote durable rejection and immunity in squamous cell carcinomas. J Immunother Cancer. (2019) 7:62. 10.1186/s40425-018-0493-9 30832732 PMC6399967

[118] Holmgaard RB Schaer DA Li Y Castaneda SP Murphy MY Xu X. Targeting the TGFβ pathway with galunisertib, a TGFβRI small molecule inhibitor, promotes anti-tumor immunity leading to durable, complete responses, as monotherapy and in combination with checkpoint blockade. J Immunother Cancer. (2018) 6:47. 10.1186/s40425-018-0356-4 29866156 PMC5987416

[119] Herbertz S Sawyer JS Stauber AJ Gueorguieva I Driscoll KE Estrem ST. Clinical development of galunisertib (LY2157299 monohydrate), a small molecule inhibitor of transforming growth factor-β signaling pathway. Drug Des Devel Ther. (2015) 9:4479–99. 10.2147/DDDT.S86621 26309397 PMC4539082

[120] Martin CJ Datta A Littlefield C Kalra A Chapron C Wawersik S. Selective inhibition of TGFβ1 activation overcomes primary resistance to checkpoint blockade therapy by altering tumor immune landscape. Sci Transl Med. (2020) 12:eaay8456. 10.1126/scitranslmed.aay8456 32213632

[121] Voron T Colussi O Marcheteau E Pernot S Nizard M Pointet AL. VEGF-A modulates expression of inhibitory checkpoints on CD8+ T cells in tumors. J Exp Med. (2015) 212:139–48. 10.1084/jem.20140559 25601652 PMC4322048

[122] Kim CG Jang M Kim Y Leem G Kim KH Lee H. VEGF-A drives TOX-dependent T cell exhaustion in anti-PD-1-resistant microsatellite stable colorectal cancers. Sci Immunol. (2019) 4:eaay0555. 10.1126/sciimmunol.aay0555 31704735

[123] Dudley JC Lin MT Le DT Eshleman JR. Microsatellite instability as a biomarker for PD-1 blockade. Clin Cancer Res. (2016) 22:813–20. 10.1158/1078-0432.CCR-15-1678 26880610

[124] Le DT Uram JN Wang H Bartlett BR Kemberling H Eyring AD. PD-1 blockade in tumors with mismatch-repair deficiency. N Engl J Med. (2015) 372:2509–20. 10.1056/NEJMoa1500596 26028255 PMC4481136

[125] Courau T Nehar-Belaid D Florez L Levacher B Vazquez T Brimaud F. TGF-β and VEGF cooperatively control the immunotolerant tumor environment and the efficacy of cancer immunotherapies. JCI Insight. (2016) 1:e85974. 10.1172/jci.insight.85974 27699271 PMC5033816

[126] Young A Mittal D Stagg J Smyth MJ. Targeting cancer-derived adenosine: new therapeutic approaches. Cancer Discov. (2014) 4:879–88. 10.1158/2159-8290.CD-14-0341 25035124

[127] Loi S Pommey S Haibe-Kains B Beavis PA Darcy PK Smyth MJ. CD73 promotes anthracycline resistance and poor prognosis in triple negative breast cancer. Proc Natl Acad Sci USA. (2013) 110:11091–6. 10.1073/pnas.1222251110 23776241 PMC3704029

[128] Nagate Y Ezoe S Fujita J Okuzakis D Motooka D Ishibashi T. Ectonucleotidase CD39 is highly expressed on ATLL cells and is responsible for their immunosuppressive function. Leukemia. (2020). 10.1038/s41375-020-0788-y. [Epub ahead of print]. 32203145 PMC7787980

[129] Bastid J Regairaz A Bonnefoy N Dejou C Giustiniani J Laheurte C. Inhibition of CD39 enzymatic function at the surface of tumor cells alleviates their immunosuppressive activity. Cancer Immunol Res. (2015) 3:254–65. 10.1158/2326-6066.CIR-14-0018 25403716

[130] Cai XY Wang XF Li J Dong JN Liu JQ Li NP. High expression of CD39 in gastric cancer reduces patient outcome following radical resection. Oncol Lett. (2016) 12:4080–6. 10.3892/ol.2016.5189 27895775 PMC5104239

[131] Chen S Fan J Zhang M Qin L Dominguez D Long A. CD73 expression on effector T cells sustained by TGF-β facilitates tumor resistance to anti-4-1BB/CD137 therapy. Nat Commun. (2019) 10:150. 10.1038/s41467-018-08123-8 30635578 PMC6329764

[132] Ryzhov SV Pickup MW Chytil A Gorska AE Zhang Q Owens P. Role of TGF-β signaling in generation of CD39+CD73+ myeloid cells in tumors. J Immunol. (2014) 193:3155–64. 10.4049/jimmunol.1400578 25127858 PMC4157098

[133] Young A Ngiow SF Barkauskas DS Sult E Hay C Blake SJ. Co-inhibition of CD73 and A2AR adenosine signaling improves anti-tumor immune responses. Cancer Cell. (2016) 30:391–403. 10.1016/j.ccell.2016.06.025 27622332

[134] Young A Ngiow SF Gao Y Patch AM Barkauskas DS Messaoudene M. A2AR adenosine signaling suppresses natural killer cell maturation in the tumor microenvironment. Cancer Res. (2018) 78:1003–16. 10.1158/0008-5472.CAN-17-2826 29229601

[135] Reinhardt J Landsberg J Schmid-Burgk JL Ramis BB Bald T Glodde N. MAPK signaling and inflammation link melanoma phenotype switching to induction of CD73 during immunotherapy. Cancer Res. (2017) 77:4697–709. 10.1158/0008-5472.CAN-17-0395 28652246

[136] Blay J White TD Hoskin DW. The extracellular fluid of solid carcinomas contains immunosuppressive concentrations of adenosine. Cancer Res. (1997) 57:2602–5. 9205063

[137] Hatfield SM Kjaergaard J Lukashev D Belikoff B Schreiber TH Sethumadhavan S. Systemic oxygenation weakens the hypoxia and hypoxia inducible factor 1alpha-dependent and extracellular adenosine-mediated tumor protection. J Mol Med. (2014) 92:1283–92. 10.1007/s00109-014-1189-3 25120128 PMC4247798

[138] Iannone R Miele L Maiolino P Pinto A Morello S. Adenosine limits the therapeutic effectiveness of anti-CTLA4 mAb in a mouse melanoma model. Am J Cancer Res. (2014) 4:172–81. 24660106 PMC3960454

[139] Mittal D Young A Stannard K Yong M Teng MW Allard B. Antimetastatic effects of blocking PD-1 and the adenosine A2A receptor. Cancer Res. (2014) 74:3652–8. 10.1158/0008-5472.CAN-14-0957 24986517

[140] Beavis PA Henderson MA Giuffrida L Mills JK Sek K Cross RS. Targeting the adenosine 2A receptor enhances chimeric antigen receptor T cell efficacy. J Clin Invest. (2017) 127:929–41. 10.1172/JCI89455 28165340 PMC5330718

[141] Young A Ngiow SF Madore J Reinhardt J Landsberg J Chitsazan A. Targeting adenosine in BRAF-mutant melanoma reduces tumor growth and metastasis. Cancer Res. (2017) 77:4684–96. 10.1158/0008-5472.CAN-17-0393 28652244

[142] Chen L Diao L Yang Y Yi X Rodriguez BL Li Y. CD38-mediated immunosuppression as a mechanism of tumor cell escape from PD-1/PD-L1 blockade. Cancer Discov. (2018) 8:1156–75. 10.1158/2159-8290.CD-17-1033 30012853 PMC6205194

[143] Hatfield SM Kjaergaard J Lukashev D Schreiber TH Belikoff B Abbott R. Immunological mechanisms of the antitumor effects of supplemental oxygenation. Sci Transl Med. (2015) 7:277ra30. 10.1126/scitranslmed.aaa1260 25739764 PMC4641038

[144] Li XY Moesta AK Xiao C Nakamura K Casey M Zhang H. Targeting CD39 in cancer reveals an extracellular ATP- and inflammasome-driven tumor immunity. Cancer Discov. (2019) 9:1754–73. 10.1158/2159-8290.CD-19-0541 31699796 PMC6891207

[145] Ma Y Adjemian S Mattarollo SR Yamazaki T Aymeric L Yang H. Anticancer chemotherapy-induced intratumoral recruitment and differentiation of antigen-presenting cells. Immunity. (2013) 38:729–41. 10.1016/j.immuni.2013.03.003 23562161

[146] Perrot I Michaud HA Giraudon-Paoli M Augier S Docquier A Gros L. Blocking antibodies targeting the CD39/CD73 immunosuppressive pathway unleash immune responses in combination cancer therapies. Cell Rep. (2019) 27:2411–25.e9. 10.1016/j.celrep.2019.04.091 31116985

[147] Fong L Hotson A Powderly JD Sznol M Heist RS Choueiri TK. Adenosine 2A receptor blockade as an immunotherapy for treatment-refractory renal cell cancer. Cancer Discov. (2020) 10:40–53. 10.1158/2159-8290.CD-19-0980 31732494 PMC6954326

[148] Zelenay S van der Veen AG Bottcher JP Snelgrove KJ Rogers N Acton SE. Cyclooxygenase-dependent tumor growth through evasion of immunity. Cell. (2015) 162:1257–70. 10.1016/j.cell.2015.08.015 26343581 PMC4597191

[149] Hamada T Cao Y Qian ZR Masugi Y Nowak JA Yang J. Aspirin use and colorectal cancer survival according to tumor CD274 (Programmed cell death 1 ligand 1) Expression Status. J Clin Oncol. (2017) 35:1836–44. 10.1200/JCO.2016.70.7547 28406723 PMC5455595

[150] Markosyan N Li J Sun YH Richman LP Lin JH Yan F. Tumor cell-intrinsic EPHA2 suppresses anti-tumor immunity by regulating PTGS2 (COX-2). J Clin Invest. (2019) 130:3594–609. 10.1172/JCI127755 31162144 PMC6715369

[151] Chalabi M Fanchi LF Dijkstra KK Van den Berg JG Aalbers AG Sikorska K. Neoadjuvant immunotherapy leads to pathological responses in MMR-proficient and MMR-deficient early-stage colon cancers. Nat Med. (2020) 26:566–76. 10.1038/s41591-020-0805-8 32251400

[152] Prendergast GC Smith C Thomas S Mandik-Nayak L Laury-Kleintop L Metz R. Indoleamine 2,3-dioxygenase pathways of pathogenic inflammation and immune escape in cancer. Cancer Immunol Immunother. (2014) 63:721–35. 10.1007/s00262-014-1549-4 24711084 PMC4384696

[153] Spranger S Koblish HK Horton B Scherle PA Newton R Gajewski TF. Mechanism of tumor rejection with doublets of CTLA-4, PD-1/PD-L1, or IDO blockade involves restored IL-2 production and proliferation of CD8(+) T cells directly within the tumor microenvironment. J Immunother Cancer. (2014) 2:3. 10.1186/2051-1426-2-3 24829760 PMC4019906

[154] Holmgaard RB Zamarin D Munn DH Wolchok JD Allison JP. Indoleamine 2,3-dioxygenase is a critical resistance mechanism in antitumor T cell immunotherapy targeting CTLA-4. J Exp Med. (2013) 210:1389–402. 10.1084/jem.20130066 23752227 PMC3698523

[155] Long GV Dummer R Hamid O Gajewski TF Caglevic C Dalle S. Epacadostat plus pembrolizumab versus placebo plus pembrolizumab in patients with unresectable or metastatic melanoma (ECHO-301/KEYNOTE-252): a phase 3, randomised, double-blind study. Lancet Oncol. (2019) 20:1083–97. 10.1016/S1470-2045(19)30274-8 31221619

[156] Messenheimer DJ Jensen SM Afentoulis ME Wegmann KW Feng Z Friedman DJ. Timing of PD-1 blockade is critical to effective combination immunotherapy with anti-OX40. Clin Cancer Res. (2017) 23:6165–77. 10.1158/1078-0432.CCR-16-2677 28855348 PMC5641261

[157] Shrimali RK Ahmad S Verma V Zeng P Ananth S Gaur P. Concurrent PD-1 blockade negates the effects of OX40 agonist antibody in combination immunotherapy through inducing T-cell apoptosis. Cancer Immunol Res. (2017) 5:755–66. 10.1158/2326-6066.CIR-17-0292 28848055

[158] Dong MB Wang G Chow RD Ye L Zhu L Dai X. Systematic immunotherapy target discovery using genome-scale *in vivo* CRISPR screens in CD8 T cells. Cell. (2019) 178:1189–204.e23. 10.1016/j.cell.2019.07.044 31442407 PMC6719679

[159] Lizotte PH Hong RL Luster TA Cavanaugh ME Taus LJ Wang S. A high-throughput immune-oncology screen identifies EGFR inhibitors as potent enhancers of antigen-specific cytotoxic t-lymphocyte tumor cell killing. Cancer Immunol Res. (2018) 6:1511–23. 10.1158/1538-7445.AM2018-4935 30242021 PMC6601346

[160] Khandelwal N Breinig M Speck T Michels T Kreutzer C Sorrentino A. A high-throughput RNAi screen for detection of immune-checkpoint molecules that mediate tumor resistance to cytotoxic T lymphocytes. EMBO Mol Med. (2015) 7:450–63. 10.15252/emmm.201404414 25691366 PMC4403046

[161] Kather JN Charoentong P Suarez-Carmona M Herpel E Klupp F Ulrich A. High-throughput screening of combinatorial immunotherapies with patient-specific in silico models of metastatic colorectal cancer. Cancer Res. (2018) 78:5155–63. 10.1158/0008-5472.CAN-18-1126 29967263

[162] Sharma P Hu-Lieskovan S Wargo JA Ribas A. Primary, adaptive, and acquired resistance to cancer immunotherapy. Cell. (2017) 168:707–23. 10.1016/j.cell.2017.01.017 28187290 PMC5391692

[163] Kluger HM Tawbi HA Ascierto ML Bowden M Callahan MK Cha E. Defining tumor resistance to PD-1 pathway blockade: recommendations from the first meeting of the SITC immunotherapy resistance taskforce. J Immunother Cancer. (2020) 8:e000398. 10.1136/jitc-2019-000398 32238470 PMC7174063

[164] Loo K Tsai KK Mahuron K Liu J Pauli ML Sandoval PM. Partially exhausted tumor-infiltrating lymphocytes predict response to combination immunotherapy. JCI Insight. (2017) 2:e93433. 10.1172/jci.insight.93433 28724802 PMC5518562

[165] Bilen MA Shabto JM Martini DJ Liu Y Lewis C Collins H. Sites of metastasis and association with clinical outcome in advanced stage cancer patients treated with immunotherapy. BMC Cancer. (2019) 19:857. 10.1186/s12885-019-6073-7 31464611 PMC6716879

[166] Daud AI Loo K Pauli ML Sanchez-Rodriguez R Sandoval PM Taravati K. Tumor immune profiling predicts response to anti-PD-1 therapy in human melanoma. J Clin Invest. (2016) 126:3447–52. 10.1172/JCI87324 27525433 PMC5004965

[167] Larkin J Chiarion-Sileni V Gonzalez R Grob JJ Cowey CL Lao CD Combined nivolumab and ipilimumab or monotherapy in untreated melanoma. N Engl J Med. (2015) 373:23–34. 10.1056/NEJMoa1504030 26027431 PMC5698905

[168] Hu-Lieskovan S Mok S Homet Moreno B Tsoi J Robert L Goedert L. Improved antitumor activity of immunotherapy with BRAF and MEK inhibitors in BRAF(V600E) melanoma. Sci Transl Med. (2015) 7:279ra41. 10.1126/scitranslmed.aaa4691 25787767 PMC4765379

[169] Ribas A Hodi FS Callahan M Konto C Wolchok J. Hepatotoxicity with combination of vemurafenib and ipilimumab. N Engl J Med. (2013) 368:1365–6. 10.1056/NEJMc1302338 23550685

[170] Minor DR Puzanov I Callahan MK Hug BA Hoos A. Severe gastrointestinal toxicity with administration of trametinib in combination with dabrafenib and ipilimumab. Pigment Cell Melanoma Res. (2015) 28:611–2. 10.1111/pcmr.12383 25996827 PMC4744965

[171] Young A Quandt Z Bluestone JA. The balancing act between cancer immunity and autoimmunity in response to immunotherapy. Cancer Immunol Res. (2018) 6:1445–52. 10.1158/2326-6066.CIR-18-0487 30510057 PMC6281171

[172] Du X Liu M Su J Zhang P Tang F Ye P. Uncoupling therapeutic from immunotherapy-related adverse effects for safer and effective anti-CTLA-4 antibodies in CTLA4 humanized mice. Cell Res. (2018) 28:433–47. 10.1038/s41422-018-0012-z 29463898 PMC5939041

[173] MenziesAMJohnsonDBRamanujamSAtkinsonVGWongANMParkJJ Anti-PD-1 therapy in patients with advanced melanoma and preexisting autoimmune disorders or major toxicity with ipilimumab. Ann Oncol. (2017) 28:368–76. 10.1093/annonc/mdw44327687304

[174] Shah NJ Al-Shbool G Blackburn M Cook M Belouali A Liu SV Safety and efficacy of immune checkpoint inhibitors (ICIs) in cancer patients with HIV, hepatitis B, or hepatitis C viral infection. J Immunother Cancer. (2019) 7:353 10.1186/s40425-019-0771-1 31847881 PMC6918622

[175] Herbaux C Gauthier J Brice P Drumez E Ysebaert L Doyen H. Efficacy and tolerability of nivolumab after allogeneic transplantation for relapsed Hodgkin lymphoma. Blood. (2017) 129:2471–8. 10.1182/blood-2016-11-749556 28270452

[176] Pai CS Simons DM Lu X Evans M Wei J Wang YH. Tumor-conditional anti-CTLA4 uncouples antitumor efficacy from immunotherapy-related toxicity. J Clin Invest. (2019) 129:349–63. 10.1172/JCI123391 30530991 PMC6307943

[177] Wu L Seung E Xu L Rao E Lord DM Wei RR Trispecific antibodies enhance the therapeutic efficacy of tumor-directed T cells through T cell receptor co-stimulation. Nature Cancer. (2020) 1:86–98. 10.1038/s43018-019-0004-z 35121834

[178] Kvarnhammar AM Veitonmaki N Hagerbrand K Dahlman A Smith KE Fritzell S. The CTLA-4 x OX40 bispecific antibody ATOR-1015 induces anti-tumor effects through tumor-directed immune activation. J Immunother Cancer. (2019) 7:103. 10.1186/s40425-019-0570-8 30975201 PMC6458634

[179] Wang H Mooney DJ. Biomaterial-assisted targeted modulation of immune cells in cancer treatment. Nat Mater. (2018) 17:761–72. 10.1038/s41563-018-0147-9 30104668

[180] Johnston RJ Su LJ Pinckney J Critton D Boyer E Krishnakumar A. VISTA is an acidic pH-selective ligand for PSGL-1. Nature. (2019) 574:565–70. 10.1038/s41586-019-1674-5 31645726

[181] Wang M Wang J Wang R Jiao S Wang S Zhang J. Identification of a monoclonal antibody that targets PD-1 in a manner requiring PD-1 Asn58 glycosylation. Commun Biol. (2019) 2:392. 10.1038/s42003-019-0642-9 31667366 PMC6814707

[182] Sun L Li CW Chung EM Yang R Kim YS Park AH. Targeting glycosylated PD-1 induces potent anti-tumor immunity. Cancer Res. (2020) 80:2298–310. 10.1158/0008-5472.CAN-19-3133 32156778 PMC7272274

